# Exploration of PVC@SiO_2_ nanostructure for adsorption of methylene blue via using quartz crystal microbalance technology

**DOI:** 10.1038/s41598-023-46807-4

**Published:** 2023-11-10

**Authors:** Safaa S. Ali, Gamal K. Hassan, Sameh H. Ismail, A. A. Ebnalwaled, Gehad G. Mohamed, M. Hafez

**Affiliations:** 1 Department of Physics, Faculty of Science, Cairo University, Giza, 12613 Egypt; 2 Department of Basic Sciences, Pyramids Higher Institute for Engineering and Technology, Giza, 12613 Egypt; 3 Water Pollution Research Department, National Research Centre, 33El-Bohouth St. (Former El-Tahrir St.), Dokki, P.O. 12622, Giza, Egypt; 4 Faculty of Nanotechnology for Postgraduate Studies, Cairo University, Sheikh Zayed Campus, 6th October City, Giza, 12588 Egypt; 5 Electronics & Nano Devices (END) Lab, Physics Department, Faculty of Science, South Valley University, Qena, 83523 Egypt; 6 Department of Chemistry, Faculty of Science, Cairo University, Giza, 12613 Egypt; 7 Nanoscience Department, Basic and Applied Sciences Institute, Egypt-Japan University of Science and Technology, New Borg El Arab, Alexandria, 21934 Egypt

**Keywords:** Environmental sciences, Chemistry, Materials science

## Abstract

Methylene blue (MB) dye is considered a well-known dye in many industries and the low concentration of MB is considered very polluted for all environment if it discharged without any treatment. For that reason, many researchers used advanced technologies for removing MB such as the electrochemical methods that considered very simple and give rapid response. Considering these aspects, a novel quartz crystal microbalance nanosensors based on different concentrations of PVC@SiO_2_ were designed for real-time adsorption of MB dye in the aqueous streams at different pHs and different temperatures. The characterization results of PVC@SiO_2_ showed that the PVC@SiO_2_ have synthesized in spherical shape. The performance of the designed QCM-Based PVC@SiO_2_ nanosensors were examined by the QCM technique. The sensitivity of designed nanosensors was evaluated at constant concentration of MB (10 mg/L) at different pHs (2, 7 and 11) and temperatures (20 °C, 25 °C, and 30 °C). From the experimental, the best concentration of PVC@SiO_2_ was 3% for adsorbed 9.99 mg of cationic methylene blue at pH 11 and temperature 20 °C in only 5.6 min.

## Introduction

Plastics play a vital role in our life as many objects which are using daily are made of different kinds of plastics such as packaging, films, covers, bags and containers, to construction, electrical and electronic applications of plastics^1^.

Polyvinyl chloride (PVC) is among the most abundant plastics worldwide, it can be used in different fields of industry including architecture, electronic, chemical engineering, pharmaceutical packaging^2,3^, transportation, while mechanical and thermal properties of PVC can be modified by incorporating some materials during PVC production^4^. Its eminent properties and good performance with low cost, great process ability, synthetic resistance and low combustibility were improved^5^.

Moreover, PVC is considered a part of plastic industry, furthermore, PVC can combine with fillers as thermal stabilizer and plasticizer, before preparing and utilizing the ideals of toughness, acid, alkali resistance and grating resistance^6^. Due to PVC little thermal stability, some materials should be added during its production to improve its thermal stability, and this was due to the unique structure and remarkable mechanical, optical, thermal and electrical properties of the used materials. By the time, PVC nanocomposites had attracted great interest because the researches have been improved the properties of PVC with loading a very low amount of nanoparticles, compared to the conventional composite materials^6–8^.

Nanoparticles additives can improve performance of polymers due to their small size, large specific area, quantum confinement effects and a strong interfacial interaction^9,10^. PVC/graphite nano-composites can be used for attenuation and electromagnetic interference (EMI) shielding^11^. PVC/CaCO_3_ nanocomposite can be used for flexibility and strength due to its novel properties^12^. These studies showed that PVC properties can be modified by adding nanoparticles and used more widely in the environment^13^. On the other side, silica nanoparticles are widely studied for many applications such as photonic crystals, chemical sensors, biosensors, nano-fillers for advanced composite materials, markers for bio imaging, substrate for quantum dots, and catalysts^14–16^. However, many wonderful reports illustrated silica uses in production of modern polymer composites, the mechanical properties and process ability of PVC filled with nano-SiO_2_ particles are widely studied^17^.

It is remarkable that polymer gives rise to new types of nanocomposite polymer electrolytes filling PVC polymers with nano-sized silica gives new types of nanocomposite polymer electrolytes. Due to optical properties of silica nanoparticles including transparency and refractive index, they are used in the research field^18^. From all the above, it is clear that, addition of nano silica to PVC matrix improved the optical and dielectric properties of the nanocomposite films^19,20^.

Population growth and industrial development have increasing pollution of the environment and needs for more energy and electricity^21^. This pollution affecting mostly water bodies and this leads to make water unsuitable for consumption^22^. One of the most common reasons of water pollution is dyes which are used in many industries, such as textiles, food, and cosmetics, and are also used for medical purposes, including as antiseptic agents^23^ or present naturally in some types of wastewater such as leachate^24^. Dyes are not only considered one of the leading water pollutants but also, they have every chance of affecting the surrounding environment in several ways^25^. Dyes pollution considered very dangerous as they have a lasting effect on exposed animals, depending on the concentration and duration of exposure and this increase bacterial growth for photosynthesis dependent bacteria and this affecting negatively on aquatic plants as bacteria will interfere with the light^26^. Dyes are used in the food industry to improve the appearance, smell, taste, color, texture, calorie content and shelf life of food. Most dyes are considered mutagenic and carcinogenic due to the presence of aromatic rings in their structures and could be present in natural and hazardous wastewater like leachate^27,28^. The presence of these dyes in food and their appearance in surface and groundwater are considered unsafe for human well-being^29^. A variety of health problems, including allergic reactions, skin irritation, gastrointestinal problems, and overactivity in children can be occurring at high doses^30,31^. Due to all these harmful effects of dyes on the health, very high regulations controlling the presence of dyes in foods are considered, and scientists are always working on developing simple and inexpensive methods for detecting and extracting dyes to lowering their contamination in the wastewater as much as they can. Detecting and identifying the content of organic dyes in food products is very hard, and, therefore, various methods are used for this purpose, including UV spectrophotometry, high-performance liquid chromatography, spectrofluorometry, electrochemical voltammetry, and mass spectrometry^32,33^. For example, methylene blue (MB) is an aromatic heterocyclic basic dye^34^ having a molecular weight of 319.85 g mol^−1^^34,35^. It is highly water-soluble, and thus forms a stable solution with water at room temperature^36–38^. Water contamination by methylene blue (MB) is a threat to human health and aquatic biota due to its toxicity, persistence, and non-biodegradability^39^. From all the above, the performance of PVC@SiO_2_ nanocomposite was investigated by measuring the removal efficiency of the methylene blue dye.

Identification of hazardous gas species and concentrations of hazardous pollutants is crucial because, in addition to polluting our living environment, toxic compounds also pose harm to human health once they reach a specific level^40–44^. To this purpose, a variety of gas and hazardous pollutants sensors, such as the quartz crystal microbalance (QCM)^41,42^, have been developed for effective environment monitoring and respiratory analysis. The Quartz Crystal Microbalance (QCM) is a physical nano-gram-sensitive device^44^ which is suited as a transducer element for electrochemical sensors because it is considered as rapid, easy to be used, highly stable and portable. QCM-based sensors can be used in detecting several analytes in very different matrix environment^45^. The QCM-based sensors can accurately detect the trace mass changes in the nano-gram range that absorbed onto the electrode of the Quartz Crystal surface in both air or liquid^44^, but using tiny molecules in making these devices make it difficult to design QCM sensors to be used continuously. So, polymeric materials can modify the surface characteristics of the QCM and hence, increase the range of QCM sensors’ applications^46,47^.

In light of this, PVC@SiO_2_ nanocomposite for QCM applications was created. atomic force microscopy) AFM), X-ray diffraction (XRD), dynamic light scattering (DLS), Brunauer–Emmett–Teller (BET) surface area, zeta-potential measurements, scanning and transmission electron microscopes (SEM and TEM), were used to characterize the nanocomposite. The QCM technology for detection of methylene blue in the aqueous solution was used to test the activity of the PVC/SiO_2_ nancomposite related to this technology. According to the findings, the PVC/SiO_2_ nanocomposite effectively could detect the methylene blue in the aqueous solution.

## Materials and methods

### Synthesis of PVC@SiO_2_ nanocomposite

Synthesis has been done by two steps; the first one synthesis of nano filler (silica nanoparticles) by sol–gel technique as used before in some literature such as Refs.^17,19,48^ and the second step was the synthesis of nanocomposite by hybrid nano silica into PVC polymer. In any case, silica nanoparticles synthesis was performed by following the gel formation method (hydrolysis of sodium silicate in acidic media using sulfuric acid). In typical synthesis, 1g of sodium silicate was dissolved in 145 ml of doubled deionized water then sulfuric acid was added drop by drop until pH becomes 2.0 and clear gel was obtained. Gel was dried under temperature of 600 °C for 2 h and finally gently milling in mortar to get fine powder of nano silica. PVC@SiO_2_ nanostructure was synthesized by dissolving 5g of PVC in 100 DHF with stirring for 5 h at 60 °C until a transparent solution was watched. The calculated weight of the prepared SiO_2_ nanoparticles (0.05, 0.1, 0.15 and 0.2g) was added to the solution under ultrasonic stirrer at room temperature (RT) for 30 min to prevent the agglomeration of the nanoparticles. Then, the synthesized 1%, 2%, 3% and 4% PVC@SiO_2_ nanocomposites were cast in glass dish of 10 cm diameters and air dried for 24 h^17,48^. During the synthesis of the nanocomposites, the conditions were well adjusted, and the silica nanoparticles were well distributed to prevent the agglomerates as shown in the following results.

### Instrumentation

X-ray diffraction (EQUINOX 1000, Thermo Scientific CO., Lafayette, CO, USA) was used to determine the composition and phase of PVC@SiO_2_ nanocomposites. Cu Kα radiation with a current of 31 mA and an applied voltage of 33 kV was used. The 2θ angles ranged between 0° to 85°, and the scan speed was adjusted to 0.1°/min. In addition, the surface charge and particle size of PVC@SiO_2_ were determined using the zeta seizer instrument (NanoSight NS500, Malvern Panalytical, Malvern, UK). The prepared samples were further examined with a TEM instrument (JEOL, JEM-2100 high-resolution, Peabody, MA, USA) to determine the morphology of PVC@SiO_2_. The PVC@SiO_2_ sample was sonicated for 20 min using an ultrasonic probe sonicator (UP400S, Hielscher, Oderstraße, Teltow, Germany) at a frequency of 55 kHz, an amplitude of 55%, and a cycle of 0.55 before TEM analysis. The dispersed mixture was then deposited in drops with a diameter of five to ten microns across a copper grid that had been coated with carbon before being subjected to TEM analysis. AFM was carried out by AFM instrument model of (5600Ls manufacture by Agilent technology, USA). The surface area and pore size of a sample were determined using BET Surface Area Analyzer manufacture by (Quanta chrome model of NOVA touch 2LX) as reported before^49^. The QCM measurements have been done using a QCM system (QCM, Q-senses, Biolin Scientific, Linthicum Heights, MD, USA) and these measurements have been done following some researches^50^.

### Application of QCM

QCM base-method depends on piezoelectric properties of quartz mineral chips in which quartz development electric charge when subjected to stress load by nanogram^50^. Detection of MB have been done by using PVC@SiO_2_ nanostructure adsorber for MB in aqueous solution which can be determined by QCM where some information about the period enough to absorb MB from solution in different temperatures and pH parameters using constant weight of MB and different concentrations of PVC@SiO_2_ nanostructure was gutted. The weight of MB adsorbed was monitored by studying the behavior of adsorbed MB on surface of PVC@SiO_2_ nanostructure with time. Twenty four QCM chips were prepared in three steps. The first step involved cleaning of QCM chips by cleaning solution consisting of 5:1:1 v/v/v solution of aqueous ammonia, H_2_O_2_, and double-distilled water) for 15 min. The second step is the precipitation of thin film of PVC@SiO_2_ nanostructure on QCM chip surface using EMITECH K450X sputter coater. In this step 10 mg of 1%, 2%, 3% and 4% of PVC@SiO_2_ nanostructure was dissolved in 20 ml DHF and coated on the surface of QCM chip by sputter coater. Finally, the QCM chips were air dried for 24 h in vacuum system. The third step is the insertion of QCM@PVC@SiO_2_ in QCM device and injecting the background doubled deionized water as electrolyte solution to blank baseline. After a stable base line, the device was now ready for the evaluation of the adsorption of MB on surface of QCM@ PVC@SiO_2_ at different temperatures and pHs^46^. Each data measurement was performed by injecting 10 mg of MB dissolved in 50 ml doubled deionized distilled water onto the surface of QCM@ PVC@SiO_2_ either at various temperatures (20 °C, 25 °C and 30 °C) or pH values (2, 7 and 11). The MB solution was then injected continuously until the frequency signal stabilized which indicating that the equilibrium of the binding interaction between the QCM@PVC@SiO_2_ and the MB had been reached.

### Consent to participate

All of the authors consented to participate in the drafting of this manuscript.

## Results and discussion

### XRD for PVC@SiO_2_ nanostructure

**Figure 1:**
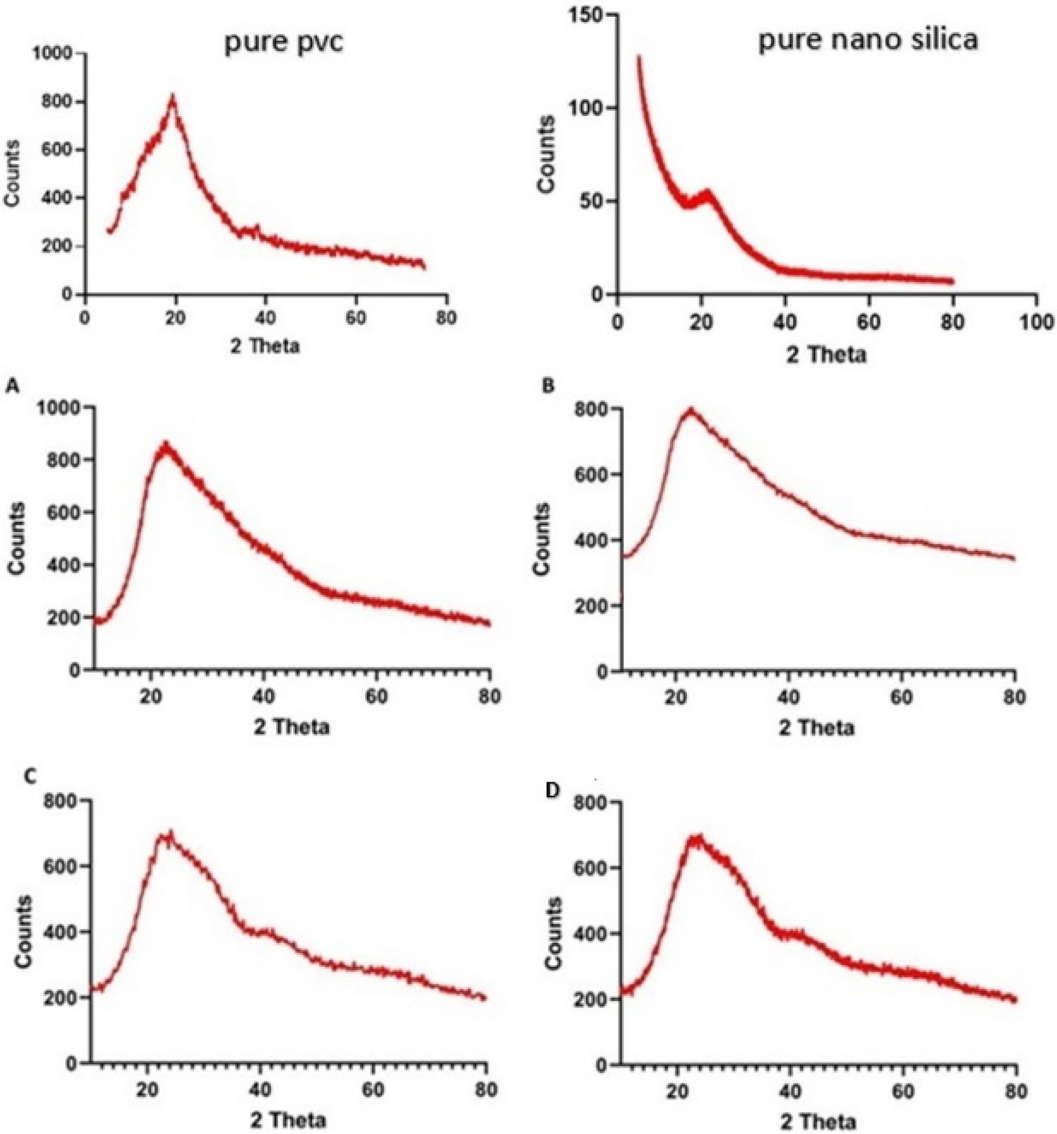
X-ray diffraction pattern for pure PVC, pure nano silica and PVC@SiO_2_ nanocomposite at different concentrations of (**A**) 1%, (**B**) 2%, (**C**) 3%, and (**D**) 4%.

X-ray diffraction (XRD) was used to characterize the sensitized nano-composite of PVC@SiO_2_ with different concentrations namely 1%, 2%, 3% and 4%. The films of PVC were loaded with the mentioned concentrations of silica oxide nanoparticles. The formed wide peak from 15 to 35∘ in all cases confirmed the formation of PVC according to many previous literature studies^51^. Furthermore, the formed nanocomposite intimated a wide and shallow peak, which confirmed the formation of an amorphous structure of the nanocomposites. This showed that the addition of silica nanoparticles causes a decrease in the degree of crystallinity and a simultaneous increase in the amorphicity of the composite (Fig. 1)^48,51^. The intensities in Fig. 1, of the nanocomposite are quite similar due to the synthesis conditions which confirmed that the homogeneity and the arrangement of the silica crystal within PVC polymer for all concentrations^48^.

### AFM for PVC@SiO_2_ nanocomposite

**Figure 2:**
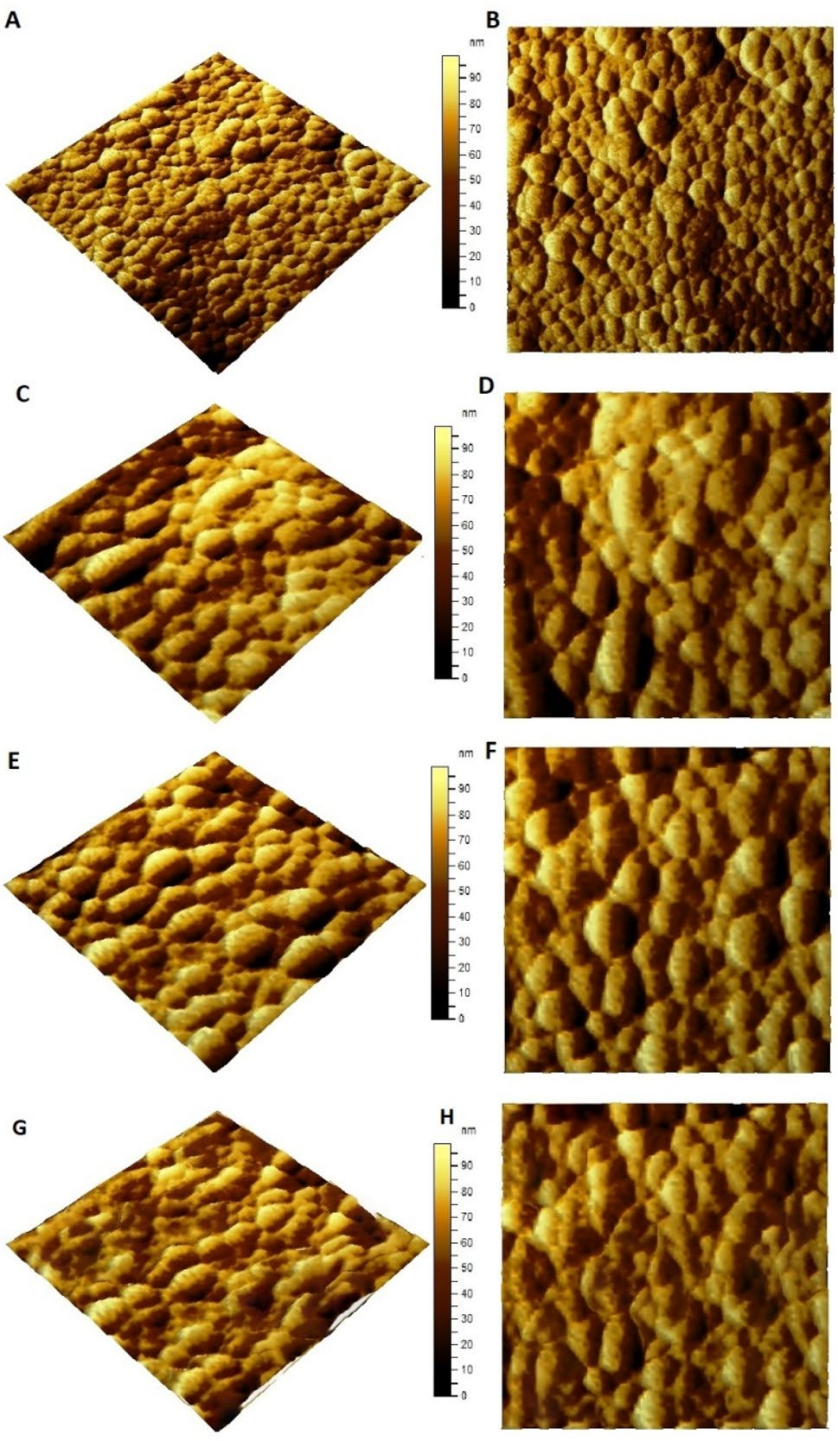
AFM for nanocomposite PVC@SiO_2_ at different concentrations of (**A**,**B**) 1%, (**C**,**D**) 2%, (**E**,**F**) 3% and (**G**,**H**) 4%.

To obtain a more in-depth insight into the shape, size, and crystalline nature of the PVC@SiO_2_ nanocomposite, AFM was employed. The 3D and 2D surface morphology images of PVC@SiO_2_ nanocomposite are consistent with what will be shown by TEM analysis. Figure 2 from -A to K- showed the AFM of the prepared nano-composite with the different concentrations. Overall, the nano-scale of the different concentrations are being cleared which confirmed that the preparation was as expected. However, the increasing of the SiO_2_ nanoparticles from 1 to 4% leads to changing in the morphology and decreasing the surface topography of the prepared nanocomposite. As a general observation, adherent and continuous silica-based films could be obtained by sol–gel on PVC substrate and moreover, increasing the concentrations to 4% lead to form the PVC@SiO_2_ nanocomposite without voids and cracks as shown in Fig. 2G. Furthermore, AFM showed that the spectral range was from 90 to 0 nm. A major problem in all nanocomposites is tendency of nanoparticles to create agglomerates at the surface of polymer as a result of their high surface area and high concentration as had been shown in a literature^52^.

### SEM and TEM for PVC@SiO_2_ nanocomposie

**Figure 3:**
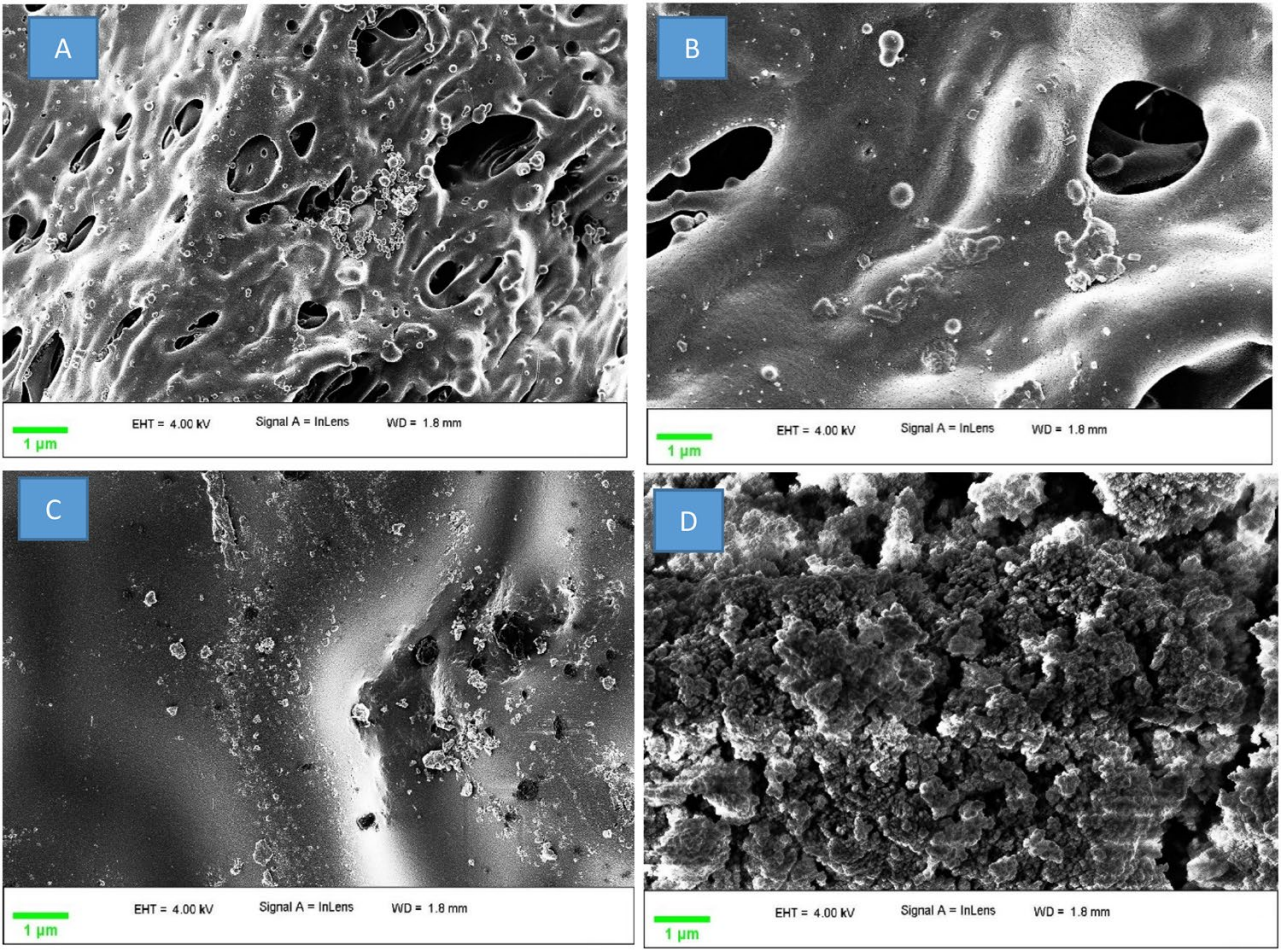
SEM for PVC@SiO_2_ nanocomposite at concentrations (**A**) 1%, (**B**) 2%, (**C**) 3% and (**D**) 4%.

Microstructure studies were carried out in order to detect voids or agglomerates that could be formed through processing steps. SEM images for PVC@SiO_2_ nanocomposies are shown in Fig. 3. It is depicted that nano silica are homogenously dispersed up to 4%. It could be observed that the smooth shape has been formed up to 3% however, the shape of the nanocomposite becomes rougher after increasing the amount of the nano-silica to 4%. This could be due to the incorporation of nano-silica particles to the PVC resulted from the various interactions between nano-silica particles and PVC molecular chains. This probably induces aggregates and agglomerates on the surface of PVC nanocomposites. As a result of this phenomenon, the surface roughness of prepared samples is increased which is in good agreement with the other sophisticated analyses. This phenomenon that the nanocomposite becomes rougher after increasing the amount of nano-silica has been observed with good agreement with other literatures^53^.

**Figure 4:**
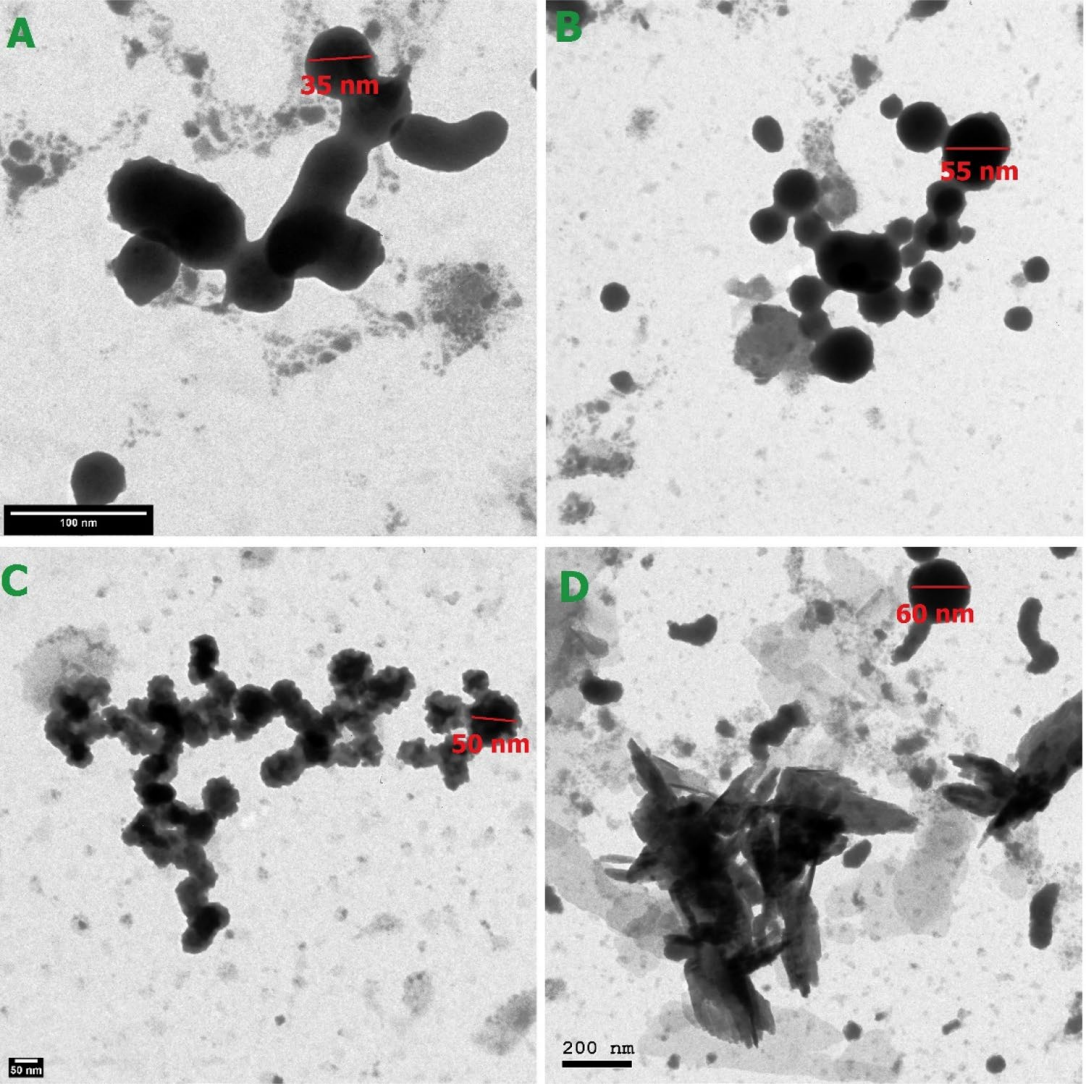
TEM for PVC@SiO_2_ nanocomposite at concentrations (**A**) 1%, (**B**) 2%, (**C**) 3% and (**D**) 4%.

TEM microscopy has been done in this study to provide detailed information about the morphology and structural characteristics of PVC@SiO_2_ nanocomposite, allowing researchers to better understand the properties and behavior of such composite materials regarding the dispersion of the nano-silica particles within the PVC matrix. Figure 4 showed that the 1% concentration of PVC@SiO_2_ nanocomposite had size of 35 nm, however, the size of PVC@SiO_2_ nanocomposite concentrations of 2%, 3% and 4% was ranged from 50 to 60 nm which confirmed the preparation of the nanocomposite in good manner.

Furthermore, Fig. 4 showed that the nano-silica particles were well-dispersed as individual and uniformly distributed particles with a consistent size and spherical-shape for the concentrations of 1%, 2% and 3%. Regarding the last concentration (4%), it can be shown that in some cases, an interaction could be occurred between nano silica and PVC and resulted in the formation of an aggregation and clustering of nano silica particles, and this can affect the properties and performance of the composite materials during their application on the treatment^54^.

### BET surface area of PVC@SiO_2_ nanocomposite

**Figure 5:**
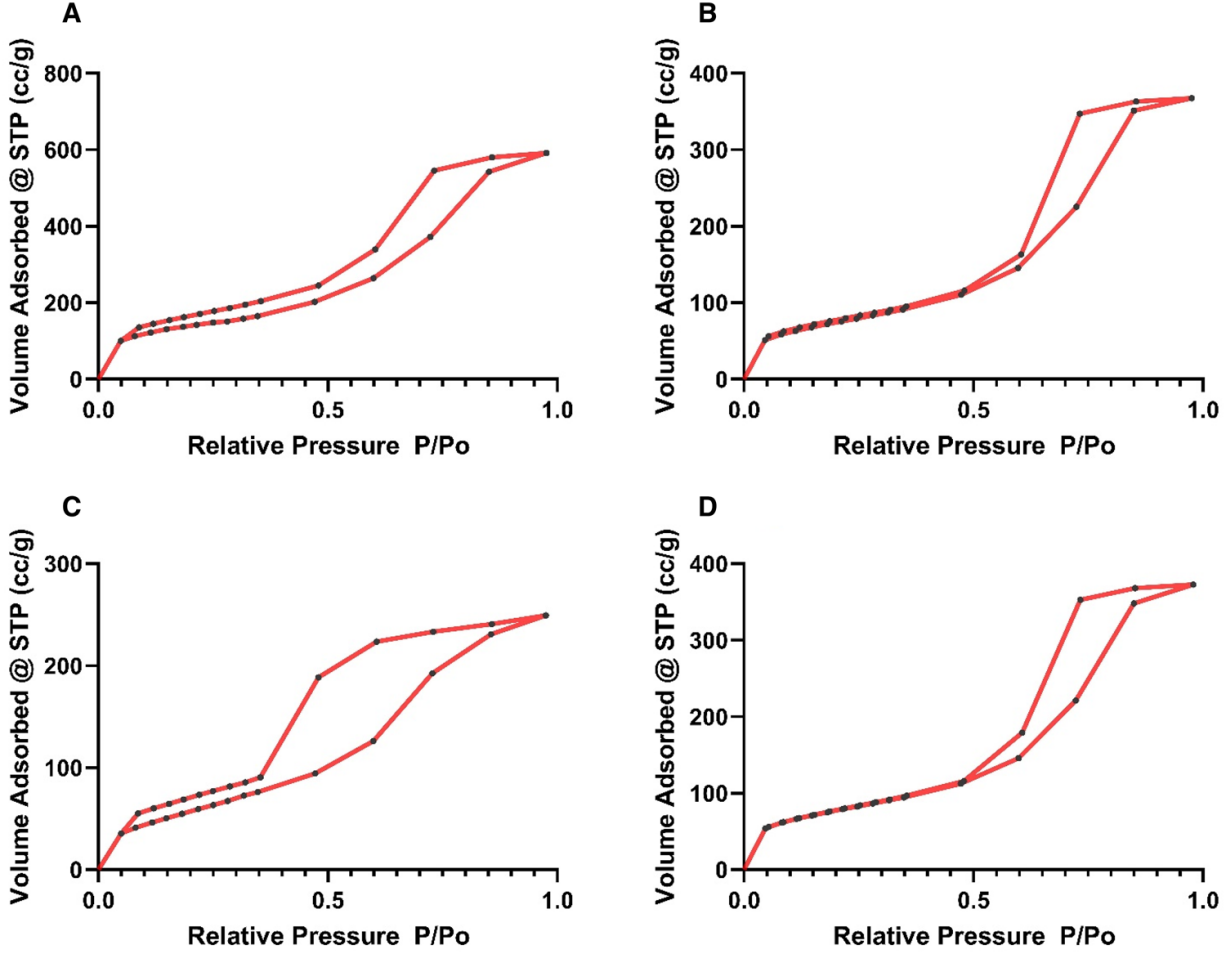
N_2_ adsorption–desorption isotherm curve at concentrations of PVC@SiO_2_ nanocomposite (**A**) 1%, (**B**) 2%, (**C**) 3% and (**D**) 4%.

Table 1 and Fig. 5 showed BET analysis of the surface area, pore size and pore volume of PVC@SiO_2_ nanocomposites (1–4%). The BET surface area for 1% PVC@SiO_2_ nanocomposite was 458.4 m^2^/g and the pore volume was 0.91 cc/g. The more loading of the nano-silica, a decrease in surface area to 277.1 m^2^/g was observed due to the filling of pores of the PVC with the nano-silica structure and this was in accordance with Thamilselvi and Radha study^55^. Silica nanoparticle loaded PVC showed further decrease in surface area to 276.9 m^2^/g and 208.9 m^2^/g for increasing the loaded nano-silica to 3% and 4%. The pore volume decreased from 0.91 cc/g to 0.57 cc/g, 0.56 cc/g, and 0.38 cc/g for PVC@SiO_2_ nanocomposite concentrations of 1%, 2%, 3%, and 4%, respectively. These results confirmed that the silica nanoparticle was loaded on PVC surfaces correctly and with a good manner. Also, the porous solid materials are classified by IUPAC into: (i) microporous materials with pore sizes up to 2.0 nm, (ii) mesoporous materials with pore sizes intermediate between 2.0 and 50.0 nm, (iii) macroporous materials with pore sizes exceeding 50.0 nm^56^. So, this confirmed that PVC@SiO_2_ nanocomposites were mesoporous materials.

**Table 1:** N_2_ adsorption–desorption isotherm for the prepared PVC@SiO_2_ nanocomposites.

PVC@SiO_2_ nanocomposites (%)	BET surface area (m^2^/g)	Pore size (nm)	Pore volume (cc/g)
1	458.4	3.8	0.91
2	277.1	4.2	0.57
3	276.9	4.1	0.56
4	208.9	3.3	0.38

### Zeta potential and DLS studies for PVC@SiO_2_ nanocomposites

**Figure 6:**
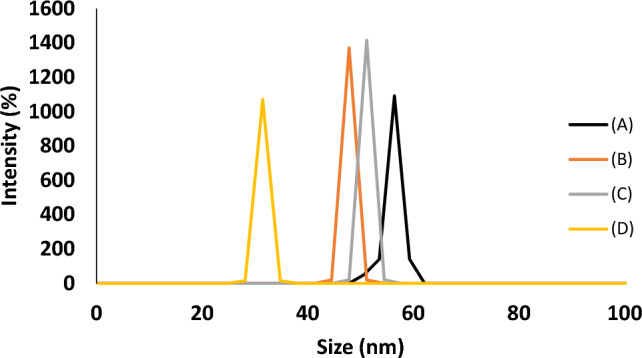
DLS for PVC@SiO_2_ nanocomposite at concentrations (**A**) 1%, (**B**) 2%, (**C**) 3% and (**D**) 4%.

**Figure 7:**
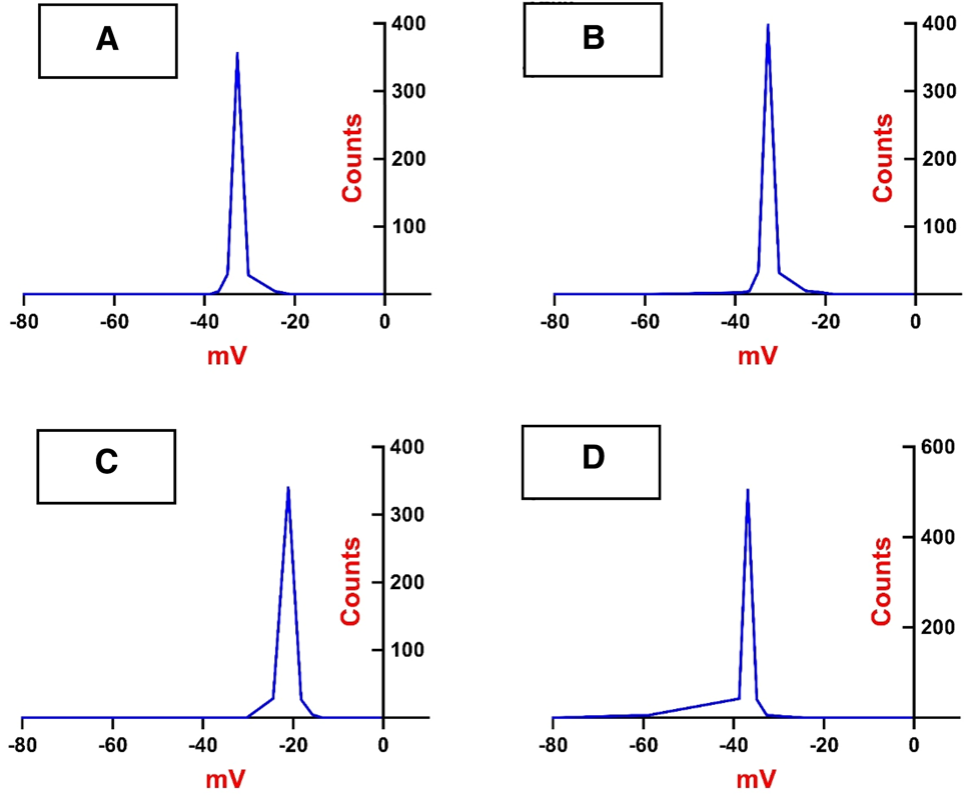
Zeta potential for PVC@SiO_2_ nanocomposite at concentrations (**A**) 1%, (**B**) 2%, (**C**) 3% and (**D**) 4%.

From Fig. 6, it can be shown that the particle size distribution, as was found by DLS analysis, showed an average particle size of 56 nm for 1%, 48 nm for 2%, 51 nm for 3% and 31 nm for 4% of PVC@SiO_2_ nanocomposites which confirmed that the prepared nanocomposites fell in the required range before being used in this study. Figure 7 showed the zeta potential that can help as a measure of the stability of a colloidal system and this analysis could be used for evaluation of the particles within a colloid of the prepared nanocomposite^57^. In this study, the prepared PVC@SiO_2_ by different concentrations was a colloid. The ZP values were approximately − 32, − 30, − 21 and − 37 mV for the prepared PVC@SiO_2_ of 1%, 2%, 3% and 4%, respectively. Zeta potentials of − 30 mV value at which colloids formed nanocomposites are commonly considered to be stabilized electrostatically and this has been confirmed by the authors of Refs.^58,59^.

### MB adsorption using QCM-based PVC@SiO_2_ nanosensors at different pH and different temperatures

The sensor based on quartz crystal microbalance (QCM) is an essential and promising sensing device for the real-time detection of dyes in aqueous solution. A QCM resonator can sensitively and precisely monitor the change in quartz resonance frequency caused by the mass adsorbed on the piezoelectric quartz crystal. The QCM-based sensor has been thoroughly investigated for detecting the trace mass changes in the nanogram range that are absorbed onto the electrode surface of the quartz crystal in air or in liquid, and it is exceedingly sensitive. For the very sensitive and precise detection of heavy metal ions in aqueous media, several QCM sensors containing layers of tiny molecules have been developed^60,61^. Due to the limitations of tiny molecules in manufacturing tools, the design of QCM sensors for use in continuous fluids is still difficult^60,61^.

**Figure 8:**
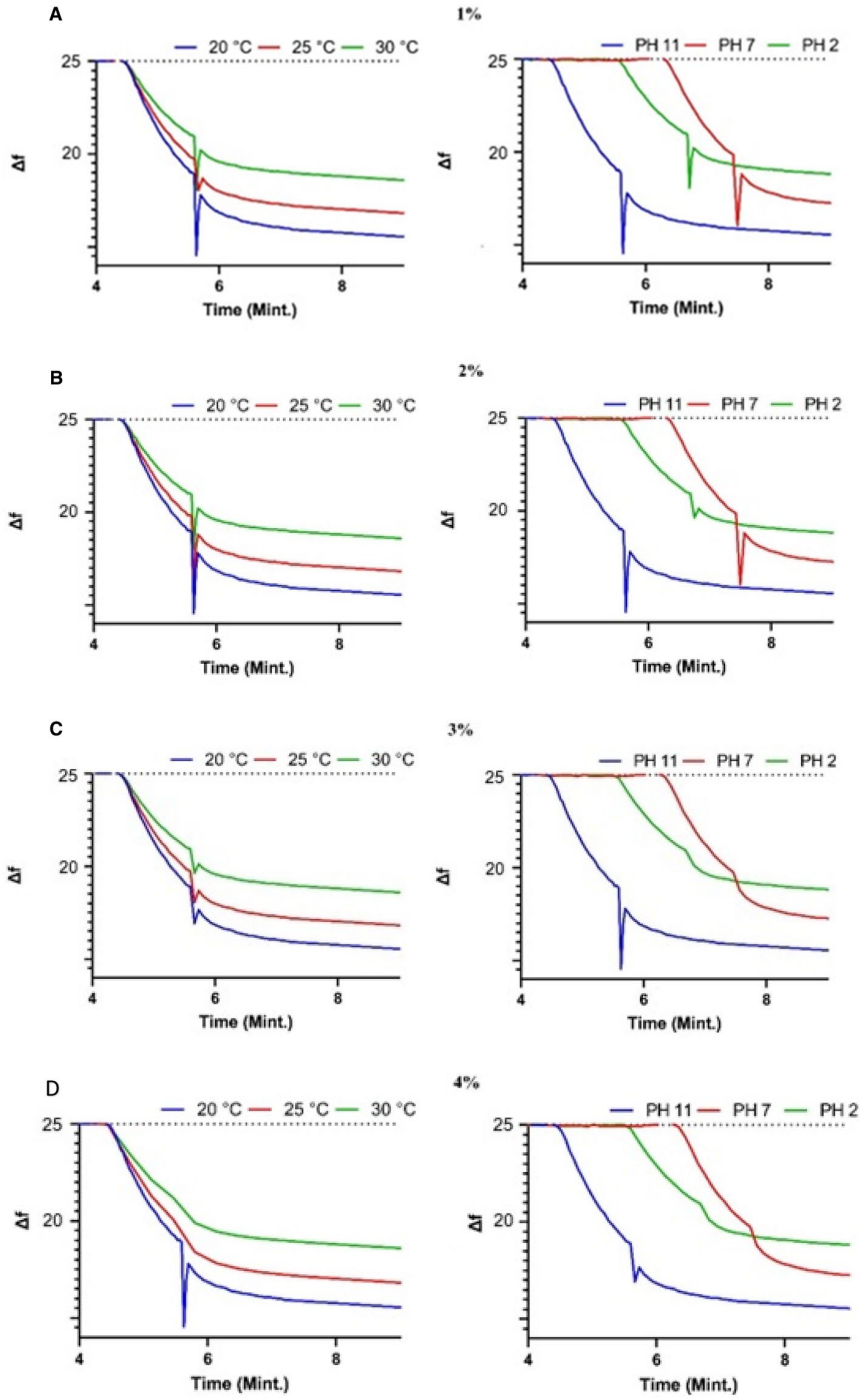
The real-time adsorption of MB dye at different pH and different temperatures for PVC@SiO_2_ nanocomposite at concentrations (**A**) 1%, (**B**) 2%, (**C**) 3% and (**D**) 4%.

**Figure 9:**
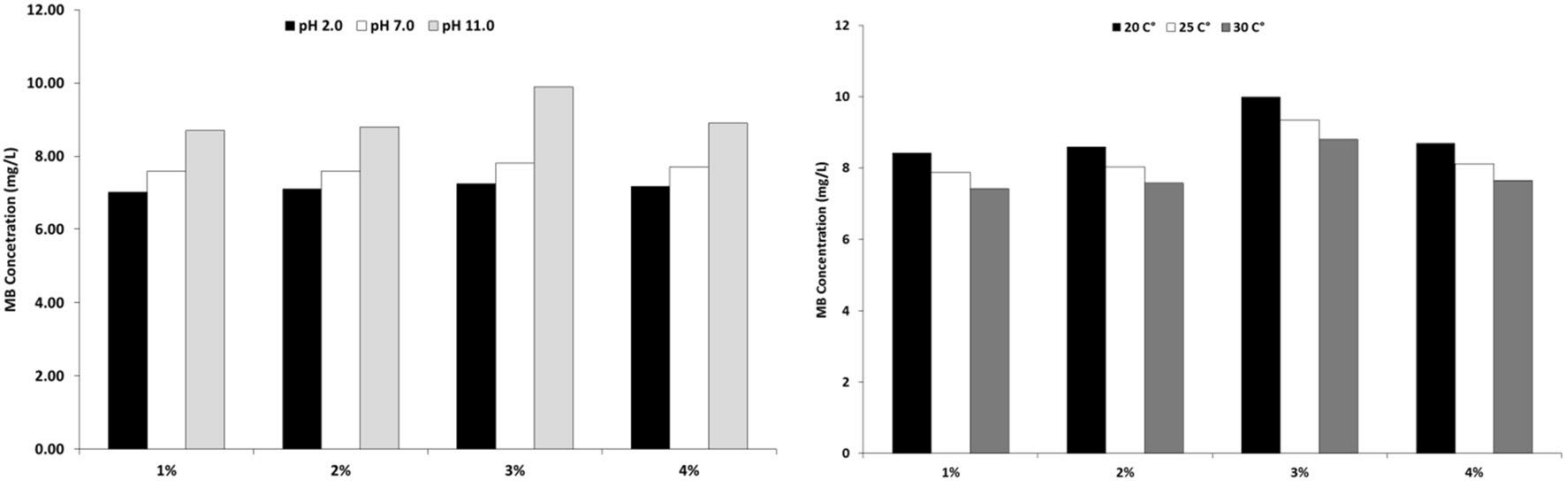
Summary chart for adsorption process of MB at different pH and different Temperature.

**Figure 10:**
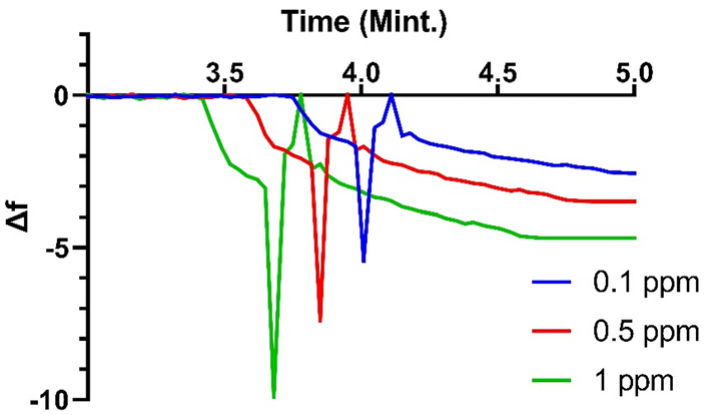
Selectivity evaluation by testing 3% PVC@SiO_2_ at pH 11 and temperature 20 °C as a sensor for MO dye using different concentrations (0.1, 0.5 and 1 ppm).

QCM technique was used for adsorption of MB cationic dye using a constant concentration of MB to illustrate the role of change in QCM@PVC@SiO_2_ concentration while changing pH value and changing temperature values as will be discussed in the following section. The QCM frequency nanogram curve of 1%, 2%, 3% and 4% PVC@SiO_2_ nanocomposite was shown in Fig. 8. This figure illustrated the increase in the amount of MB adsorbed by increasing pH value from 2.0 to 7.0 and 11.0. This increase in the adsorption value can be accounted for the increase in the charge density in surface of QCM@PVC@SiO_2_ which increased the interaction between cationic MB dye and PVC@SiO_2_ nanocomposite. However, according to QCM data all samples succeeded in absorbing 10 mg of MB dye at all pH values but the time of removal of MB dye from solution was different as shown in Fig. 8. All PVC@SiO_2_ dosages (1%, 2%, 3% and 4%) showed super speed absorption and removal for MB dye from solution at the time from 5 to 7 min whereas the previous work utilized 100mg/L of MB showed adsorption of 7.25 to 6.67 mg of MB in 90 min using nanotube and nanosheet of titania^62^. However, frequency change (Δf) of (1%, 2%, 3% and 4%) PVC@SiO_2_ curves for different pH illustrated the best mass of MB adsorbed on QCM@PVC@SiO_2_ surface at pH 11, 2 and 7, respectively. The experimental results illustrated decrease in the time and rate of adsorbed cationic MB dye on the surface of QCM@PVC@SiO_2_ by increasing temperature due to weakness of electrostatic interaction by increasing temperature. Frequency change (Δf) of (1%, 2%, 3% and 4%) PVC@SiO_2_ curves for different temperatures illustrated the best mass of MB adsorbed on QCM@PVC@SiO_2_ surface at temperatures 20 °C, 25 °C and 30 °C, respectively. However, from our experimental data, the best concentration of PVC@SiO_2_ nanocomposite was 3% for adsorption of 9.99 mg of cationic methylene blue dye at pH 11 for temperature 20 °C in only 5.6 min and a summary chart can conclude our findings as shown in Fig. 9. The reason for that the best concentration was 3% of PVC@SiO_2_ may be revealed to that after increasing the amount of SiO_2_ to 4% on the PVC surface, a formation of an aggregation and clustering of nano-silica particles has been happen, and this can affect the properties and performance of the composite materials. The selectivity of the PVC@SiO_2_ sensor towards methylene orange (MO) dye was evaluated by QCM measurements as shown in Fig. 10. PVC@SiO_2_ with 3% concentration was coated onto QCM electrodes by drop casting method. Solutions of MO of different concentrations of (0.1 ppm, 0.5 ppm and 1 ppm) were flowed over the sensor surface at a flow rate of one mL/min for 7 min for every one with recording of Δf. With 0.1 ppm MO, a small Δf of − 6 Hz was observed indicating MO binding to the sensor surface. When the concentration was increased to 0.5 ppm, a higher frequency change of − 7 Hz occurred due to more deposition of MO. At 1 ppm MO, the frequency further dropped to − 10 Hz as more binding sites were occupied. After each MO injection, buffer was flowed which resulted in an increase in frequency back to baseline. This suggests the bound MO was removed from the sensor surface demonstrating the reversible deposition-leaching cycle. In contrast, a control experiment was performed with methylene blue (MB) dye under identical conditions. While MB also showed concentration dependent frequency decreased, the frequency did not return to baseline on buffer injection. This indicates irreversible binding of MB without leaching. The QCM results confirm the selective detection of MO by the PVC@SiO_2_ sensor with showing a reversible MO binding that was dependent on concentration. The selectivity can be attributed to the hydrogen bonding and electrostatic interactions between MO and the SiO_2_ and PVC components.

### Mechanism of QCM sensing

The PVC@SiO_2_ nanocomposite film demonstrated greater sensitivity may be due to the presence of PVC and SiO_2_ NPs within the hydrogel matrix. By incorporating PVC and SiO_2_ NPs into the polymer matrix, the sensing properties of the PVC@SiO_2_ nanocomposite were improved^63^. Therefore, the combination of PVC and SiO_2_ NPs can complement each other and effectively produce nanocomposites film with excellent sensing performance owing to the large specific surface area and many alkenyl, chloride and oxygen-containing functional groups of the PVC@SiO_2_ hybrid system^63^. As a result, it serves as a good sensor platform characterized by high sensitivity due to its ability to enhance electron transfer when immobilized into QCM electrodes. It can be concluded that physisorption is the primary process that drives MB dye adsorption by PVC@SiO_2_ NPs are the most significant contributor to this process. When in solution, PVC atoms tend to become protonated and acquire a negative charge, which enables them to attract positively charged of MB dye molecules through electrostatic forces. While the SiO_2_ NPs assisted by increasing the loading area and the surface area for PVC@SiO_2_ NPs, which also resulted in the removal of some MB molecules through intercalation. The loading area and the surface area could reach at the maximum to adsorb the maximum amount of the methylene blue depending on the crystallinity and the homogeneity of the materials and after the maximum point, adding any amount of SiO_2_ on the PVC will lead to different shape and change in the crystallinity and could lead to decreasing the adsorption capacity for the material. In this study, the optimum concentrations of the SiO_2_ on the PVC was 3% and after this percent, the efficiency of the materials in the adsorption capacity will decrease as happen in some modifications that used for adsorption process and confirmed that after increasing the concentrations of the nanoparticles, the adsorption process could be decreased^64,65^.

## Conclusion

In this work, novel QCM-based PVC@SiO_2_ nanosensors with different concentrations have been developed for adsorption of MB dye from the water streams. X-ray diffraction (XRD) was used to characterize the sensitized PVC@SiO_2_ nanocomposite with different concentrations namely 1%, 2%, 3% and 4%. The films of PVC were loaded with the mentioned concentrations of silica oxide nanoparticles. The formed wide peak from 15 to 35∘ in all cases confirmed the formation of PVC. The concentration of PVC/nano silica concentrations of 1% was in the size of 35 nm and the size of PVC/nano silica concentrations of 2%; 3% and 4% was ranged from 50 to 60 nm which confirmed the preparation of the nanocomposite was in the good manner. The sensitivity of designed nanosensors was evaluated at constant concentration of MB (10 mg/L) at different pHs and temperatures (20 °C, 25 °C, and 30 °C). From our experimental, the best concentration of PVC@SiO_2_ nanocomposite was 3% for adsorption of 9.99 mg of cationic methylene blue dye at pH 11 for temperature 20 °C in only 5.6 min.

## Data Availability

The datasets used and/or analyzed during the current study are available from the corresponding author upon reasonable request.
